# Supplementary material for: Towards the Neuroevolution of Low-level artificial general intelligence
Source: Front Robot AI. 2022 Oct 14;9:1007547. doi: 10.3389/frobt.2022.1007547 (PMC9613950; doi:10.3389/frobt.2022.1007547)
Supplement: Supplementary file 1 [file DataSheet1.PDF]

Supplementary Material

1 CART-POLE BALANCING ENVIRONMENT POLE SIZES

The cart-pole balancing tasks have five different pole sizes. Three are used for training and two are for testing. We use the poles sizes 0.5 (default), 0.3, and 0.7 for training; and, the sizes 0.4, and 0.6 for testing. Fig. 1 shows all those different environment conditions.

2 CART-POLE BALANCING ENVIRONMENT OBSERVATIONS TO FIRING RATE

The four observations from the cart-pole balancing task are converted to firing rates of three neurons. Neurons #1 and #3 use $\mathcal{F}_{sigmoid}$, while neuron #2 uses $\mathcal{F}_{Gaussian}$ (the equations are explained in the manuscript). For cart position, cart velocity, and pole angular velocity, the parameters are shown in Tab. S1. This is depicted in Fig. 2a. For pole angle, the parameters are presented in Tab. S2. These three functions are depicted in Fig. 2b.

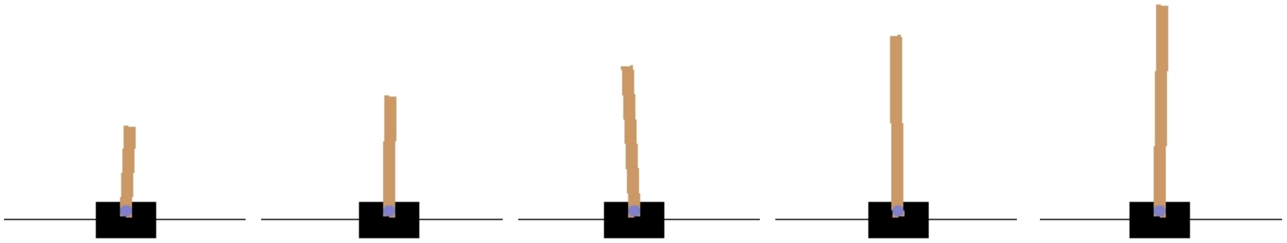

Figure 1a. Size=0.3 **Figure 1b.** Size=0.4 **Figure 1c.** Size=0.5 **Figure 1d.** Size=0.6 **Figure 1e.** Size=0.7

Figure 1. The different pole sizes for the cart-pole balancing task.

Neuron	Function	Parameters
#1	$\mathcal{F}_{sigmoid}$	$w = -2.5, z = -0.6$
#2	$\mathcal{F}_{Gaussian}$	$\mu = 0.0, \sigma = 0.4$
#3	$\mathcal{F}_{sigmoid}$	$w = 2.5, z = 0.6$

Table S1. Parameters of $\mathcal{F}_{sigmoid}$ and $\mathcal{F}_{Gaussian}$ for cart position, cart velocity, and pole angular velocity.

Neuron	Function	Parameters
#1	$\mathcal{F}_{sigmoid}$	$w = -60.0, z = -0.05$
#2	$\mathcal{F}_{Gaussian}$	$\mu = 0.0, \sigma = 0.05$
#3	$\mathcal{F}_{sigmoid}$	$w = 60.0, z = 0.05$

Table S2. Parameters of $\mathcal{F}_{sigmoid}$ and $\mathcal{F}_{Gaussian}$ for pole angle.

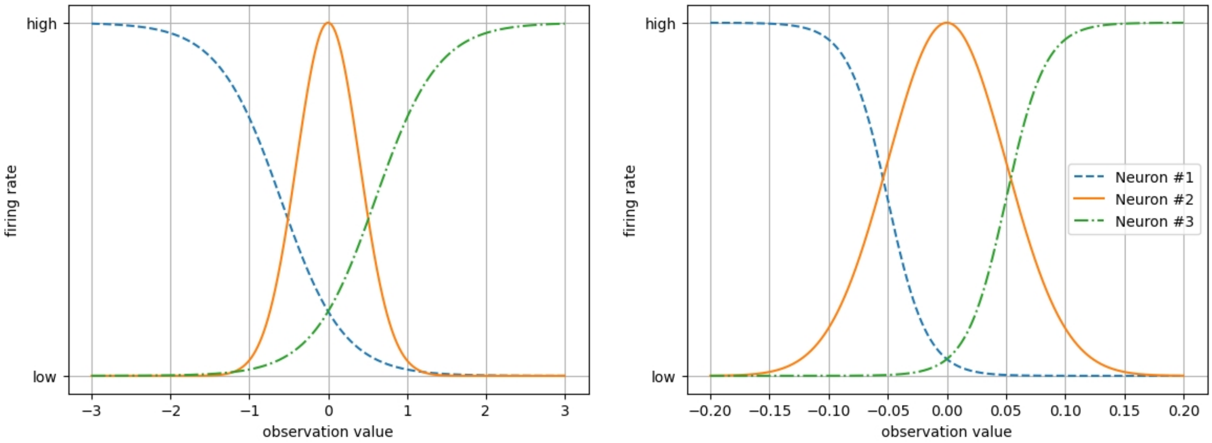

Figure 2a. Cart position, cart velocity, and pole angular velocity

Figure 2b. Pole angle (in radians)

Figure 2. Converting observation values to firing rate of the three input neurons.
